# Emergence of *Theileria* species in ticks from free-ranging domestic animals in Raymond Mhlaba local municipality, South Africa

**DOI:** 10.1016/j.heliyon.2022.e09085

**Published:** 2022-03-10

**Authors:** Benson Chuks Iweriebor, Kayode Olayinka Afolabi, Ayabulela Nqoro, Larry Chikwelu Obi

**Affiliations:** a Sefako Makgatho Health Sciences University, Ga-Rankuwa, Pretoria, South Africa; b Department of Biological Sciences, Anchor University Lagos, Nigeria

**Keywords:** Tick-borne parasites, Protozoan, Domestic animals, South Africa

## Abstract

Ticks infestation and diseases associated with it, are becoming a major life threatening concern to wildlife, domesticated animals and human health in general. Besides causing skin damage, ticks infestations have become a growing burden in food security and transmission of multiple pathogens. There is paucity of data on the occurrence of etiologic agents of tick-borne diseases in the Eastern Cape Province South Africa. We therefore carried out a molecular surveillance on *Babesia* and *Theileria* species in ticks obtained from livestock in Raymond Mhlaba District Municipality of the Province. A total of 962 ticks were collected and were morphologically identified and processed for DNA extraction using commercial DNA extraction kit. The extracted DNA samples were used to molecular identification of the ticks, and also to assess the occurrence of the *Babesia* and *Theileria* spp by PCR using genus specific primers. Positive amplicons obtained were sequenced, processed and characterised using appropriate bioinformatics tools. The molecular and morphological identifications of ticks obtained from the domestic animals in the study areas revealed that they belong to three different genera namely: *Haemophalis, Rhipicephalus,* and *Amblyomma* in ascending order of their abundance. Furthermore, the DNA of *Theileria* spp. was detected from 10 out of 962 ticks screened, with an overall infection of about 1% for *Rhipicephalus* spp., while none of the ticks was positive for *Babesia* spp. The phylogenetic analysis of the 10 theilerial sequences showed that nine (9) clustered distinctly within the *T. orientalis* complex clade, while only one (1) sequence formed a cluster with reference sequences of *T. velifera*. The findings from this study therefore expand the knowledge on recent emergence of *Theileria* spp. in livestock reared in the study area. This calls for an urgent effort in curbing the further spread of the pathogens in the area and beyond.

## Highlights

• Distribution of ticks' species in domestic animals was described.
• Emergence of theilerial parasites in ticks from free-ranging domestic animals was observed
• *Rhipicephalus* spp. of ticks serve as the major transmission vector for *Theileria* spp. in the Eastern Cape Province, South Africa

## 1 Introduction

Tick-borne zoonotic diseases are emerging public health concern globally with increased mortality among wildlife and domestic animals in general (Titcomb et al., 2017; Vesco et al., 2011). Ticks are known to be external obligate parasites, ranking second to mosquitoes in transmitting disease causing agents. They are proficient in spreading multitudes of bacterial, protozoan and viral pathogens of zoonotic importance. They constitute an increasing burden for human and animal health in a bid to survive and complete their life cycles, through the sucking of blood of their vertebrate hosts, including mammals, birds, and amphibians (Estrada-Peña et al., 2015; Mitchell et al., 2016).

Piroplasm parasites of the genera *Theileria* and *Babesia* are tick-transmitted intracellular protozoan pathogens of wild animals and domestic ruminants, and they both belong to the phylum *Apicomplexa,* a relatively large and complex group of eukaryotic organisms. *Theileria* and *Babesia* are closely related genetically as the two genera are found in the family *Piroplasmoridae;* however, they differ in their developmental stages within the host leukocytes. Though the species of *Theileria* have been reported in numerous mammalian hosts, recently, more of the *Theileria* spp*.* described were detected in ruminants, and have been identified as significant disease agents of livestocks, especially in tropical and sub-tropical regions of the world (Morrison, 2015; Aouadi et al., 2017). Presently, there are several species of *Theileria* that cause diseases in domesticated animals across the globe. Each species appears restricted to a given geographical area where they cause the disease called theileriosis in animals. The pathogenic species include: *T. parva, T. annulata, T. mutans, T. taurotragi, T. velifera, T. orientalis/sergenti/buffeli complex, T. hirci,* and *T. separate*. In Africa, *T. orientalis*, *T. buffeli* and *T. velifera* are widely distributed in many countries and are transmitted by different tick vectors (Moumouni et al., 2015; Mohammadi et al., 2017; Perveen et al., 2021).

Recently, there has been a re-evaluation in the classification of *Theileria* spp. due to variability observed in the major piroplasm surface protein (MPSP) gene through sequence analysis (Watts et al., 2016). MPSP gene sequencing analysis has established that *T. orientalis* complex should be a designated name for the *T. orientalis/sergenti/buffeli* group, which are generally non-transforming, benign, and cosmopolitan parasites detected in different regions of the world (Watts et al., 2016; Swilks et al., 2017). However, its taxonomic position has been argued for many years (Watts et al., 2016). Even though *T. orientalis* is usually regarded as non-pathogenic parasite, recently, it has been associated with clinical conditions such as disinclination to walk, weakness, and early abortion, although they are non-specific clinical outcomes of infection with *T. orientalis* (Watts et al., 2016).

In developing countries where there are limited resources, tick infestation and prevalence of tick-borne theilerial disease could be more severe (De Castro, 1997; Berggoetz et al. 2014). The Eastern Cape Province of South Africa is one of the places where tick infestation poses a major challenge, especially for small-scale farmers (Moyo and Masika, 2009). The Province is characterised with agricultural activities, and has large number of domestic animals that are kept in close vicinity to homes and nature game reserves that are located in the area. Hence, this could lead to spontaneous spread of diseases from one animal to another and ultimately to humans. Furthermore, there are limited reports on the surveillance of tick-related bacterial and protozoan pathogenic organisms in the region.

Despite the broad studies conducted in South Africa on ticks and tick-borne infections in different hosts (Horak, 1999; Chaisi et al., 2011; Berggoetz et al., 2014; Ringo et al., 2018; Bhoora et al., 2020), there is insufficient information concerning the prevalence of tick-related pathogens in the Raymond Mhlaba local Municipality of Eastern Cape Province, South Africa. This study was therefore conducted to fill the knowledge gap and to document the likely occurrence of tick-associated *Theileria* and *Babesia* in the study area.

## 2 Materials and methods

### 2.1 Ethical consideration

Prior to the commencement of this study, ethical approval was obtained from the University of Fort Hare (Alice, South Africa) Ethics Committee. Upon arrival at the sampling sites, permission was equally obtained from the farmers before ticks were collected from their livestocks. The collection of ticks was done with the assistance of various veterinary and animal health officers of the Eastern Cape Department of Agriculture and Forestry who are in-charge of handling and giving medical treatment to the farm animals.

### 2.2 Study area description

This research work was carried out from May 2017 to September 2018 at the Raymond Mhlaba local municipality area, located in the Eastern Cape, South Africa. The investigation was carried out at Debe (32.836°S 27.154°E), and Fort Beaufort (32° 47′ 0″ S, 26° 38′ 0″ E), both under the local municipality. Due to large land mass, domestic animals roam freely and are kept very close to homes. The study sites share boundaries with the Great Fish River Nature Reserve (one of the natural reserves in the Eastern Cape Province of South Africa, lies midway between King Williams Town and Grahamstown) that boast of wild mammals such as black wildebeest (*Connochaetes gnou)*, wild boars (*P. porcus koiropotamus)*, antelopes (*Aepyceros melampus)*, chacma baboons (*Papio ursinus ursinus)*, reedbucks (*Redunca arundinum*), and several rodent species.

### 2.3 Tick collection and morphological characterisation

Ticks sample were manually collected from livestocks including cattle, sheep, goats, and horses with the aid of forceps, and were transferred into 50 mL Nalgene tubes containing 70% ethanol. The samples were carried to the laboratory of Applied and Environmental Microbiology Research Group situated at the University of Fort Hare, South Africa for further study. The ticks were separated on the basis of the animal hosts and locations from which they were collected, as previously done by Iweriebor et al. (2017). Ticks were identified using morphological criteria and appropriate taxonomic keys (Walker et al., 2005). The ticks were sorted according to species, collection site, and stage of development.

### 2.4 PCR-based identification of ticks and detection of the protozoan DNA

DNA extraction from the individually minced ticks' sample was performed using the Zymoresearch Quick DNA universal kit in strict compliance to the stipulated instructions from the manufacturer. Each tick specimen was screened using PCR for tick identification and subsequently for detecting the presence of *Theileria* and *Babesia* spp*.* The confirmation of tick species was done by PCR targeting the mitochondrial 12S rRNA gene using the forward primer 85F–12S [5′-TTAAGCTTTTCAGAGGAATTTGCTC-3′] and the reverse primer 2225-12S [5′-TTTAAGCTGCACCTTGAC TTAA-3′] as described by Pesquera et al. (2015). Also, the molecular screening for tick-borne protozoan was carried out by using primers that specifically target a fragment of the 18S rRNA genes according to Jefferies et al. (2007). Two rounds of PCR with two sets of oligonucleotides targeting 18S ribosomal RNA (rRNA) gene for both species, previously reported by Jefferies et al. (2007) was adopted for this study. For the first round of PCR, forward primers (5′-GGCTCATTACAACAGTTATAG-3′) and reverse primers (5′ CCCAAAGACTTTGATTTCTCTC3′] to generate 930bp, while for the second round of PCR forward primers (5′-CCGTGCTAATTGTAGGGCTAATAC-3′) and reverse primers (5′- GGACTACGACGGTATCTGATCG-3′) to generate 800bp were used, and the cycling protocols as described by the referenced authors were adopted for the polymerase chain reactions. Amplicon verifications was done in 1% agarose gel electrophoresis for 45 min at 124 amps in 0.5X TBE buffers stained with ethidium bromide and visualized in Bio-Rad trans-illuminator. For the detection of contamination or false positive, a negative tube containing all the constituents of the PCR minus sample was used.

### 2.5 Sequencing of DNA and analyses

The amplified positive PCR products were bi-directionally sequenced at a commercial DNA sequencing facility at Stellenbosch University, South Africa using the primer pairs that were employed for the amplification of the respective fragments, while sequence editing and generation of consensus sequences were achieved using Geneious Prime 2019.0.3 version. The nucleotide sequences obtained were used for homology search using the BLAST 2.0 programme, hosted in the National Centre for Biotechnology Information (NCBI) database (www.ncbi.nlm.nih.gov/blast/Blast.cgi), in which the comparison of the edited sequences was done with previously characterized reference sequences deposited in the NCBI GenBank (Altschul et al., 1990). Sequence datasets for the positive samples were published in NCBI GenBank with the Accession number MW647866 to MW647875 for RM-SA1 to RM-SA10. Phylogenetic analysis of the data sets was conducted by using the Neighbor-Joining (NJ) method as implemented in MEGA6 (Tamura et al., 2004) with reference sequences in the GenBank (Supplementary Table 1). The reliability of the reconstructed phylogenetic tree was determined using the bootstrapping method calculated based on 1,000 replicates of the alignment, while the Jukes-Cantor parameter was used as a nucleotide substitution model.

### 2.6 Statistical analysis

The Microsoft excel and IBM SPSS version 20.0 were used for statistical analyses, using p < 0.05 as statistical significance. The student t-test was used to determine the distribution of ticks from the two sampling locations; while, One-Way ANOVA was used to determine the distribution of tick species in the sampled animals.

## 3 Results

### 3.1 Prevalence of tick species

**Figure 1:**
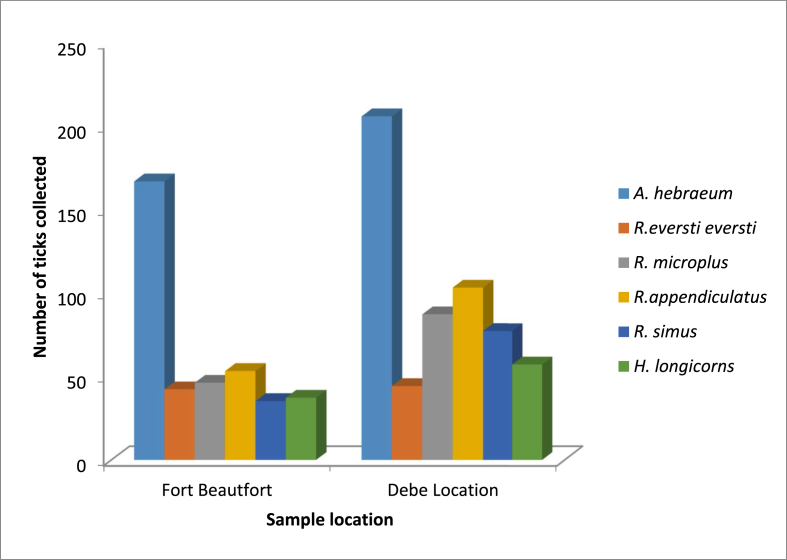
The relative abundance of tick species in the two study sites.

**Figure 2:**
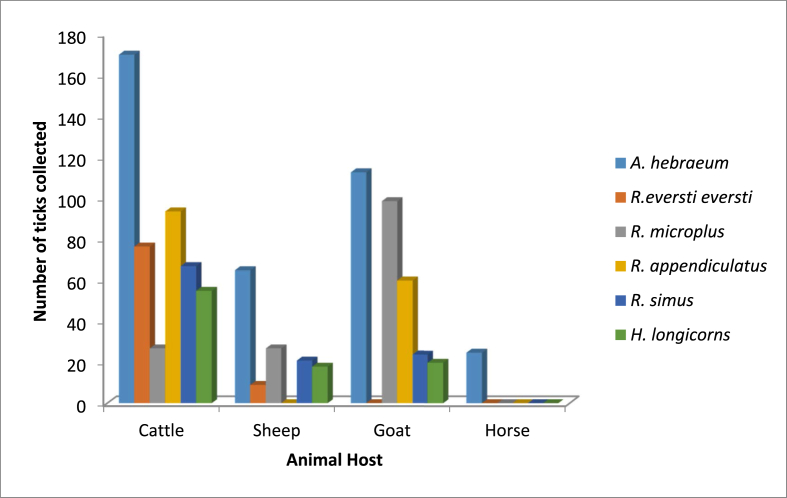
Distribution of various tick species collected from different livestock at the two study sites.

A total of 962 adult ticks were collected from the designated localities in the stipulated study area. The morphological and molecular identification showed the presence of six tick species that belong to three different genera namely *Amblyomma, Rhipicephalus*, and *Haemaphysalis.* Three hundred and eighty-two (39.7%) of the identified tick species were from Fort Beautfort; while, 60.3% (580/962) were obtained from Debe. Precisely, the significant order of dominance of the tick species are as follow: *A. hebraeum* (38.8%, n = 373), *R. appendiculatus* (17.4%, n = 167), *R. microplus* (13.6%, n = 131), *R. simus* (11.6%, n = 112), *H. longicornis* (9.7 %, n = 93) and *R. eversti eversti* (8.9 %, n = 86) (P < 0.05). Sequences derived from the ticks were only used for BLAST search and not for phylogenetic analysis because they are just 147bp in length thus giving poor phylogenetic signals. In the two study sites, *A. hebraeum* was the most prevalent tick species, while the most common and significant host is cattle (P < 0.05) (Figures 1 and 2). The two designated sites were compared based on the relative abundance of ticks collected.

### 3.2 Detection and prevalence of theileria species

A total of 10 (1%) *Theileria* spp*.* were detected from 962 tick samples that were subjected to molecular screening in this study. Incidentally, none of the tick samples came out positive for *Babesia.* The BLAST analysis showed that 90% (9/10) of the theilerial sequences have 99.8–100% identity to several other reference of *T. orientalis, T. buffeli* and *T. sergenti* sequences from the GenBank. Precisely, the sequence RM-SA1 to 9 (with the exception of the sequence RM-SA6 that showed 99.9% identity) showed a 100% identity to reference *T. orientalis* (MH208633), *T. buffeli* (Z15106) and *T. sergenti* (GU143087) sequences from China, South Africa and Taiwan respectively. Furthermore, only 1 out of 10 theilerial sequences (RM-SA10) showed high similarity to reference *T. velifera* sequence LC431549 from Saudi Arabia (99%), and 98.6% similarity with JN572705 and KU206302 from South Africa and Uganda respectively.

Notably*,* the theilerial sequences were only detected in *Rhipicephalus* tick species. Precisely, 4.7% (4/86) of the *R. eversti eversti* ticks obtained from cattles were infested with *T. orientalis* complex. Also, 3.1% (4/131) of *R. microplus* ticks from cattles equally possessed *T. velifera* (1/131) and *T. orientalis* complex (3/131). Lastly, only one *R. microplus* tick each from goat and sheep harboured the *T. orientalis* complex. The overall prevalence of *Theileria* spp. in ticks collected from cattle was 80%, while 20% prevalence was recorded for ticks from goats and sheep. Hence, it could be clearly indicated that cattle are the predominant host of *Theileria* in the study area (Table 1), probably due to high number of cattle sampled compared to other domestic animals.

**Table 1:** Prevalence of *Theileria* spp. in different tick species on animals collected from Debe location.

Animal host	*Ticks &* Theileria *spp. found in them*	No. of theilerial-positive tick samples (Strain name for the theilerial sequence in GenBank)	Percentage of theilerial-positve tick spp.	Percentage of Theilerial-positive ticks found in each host
Cattle	***R. eversti eversti***	4/86 (RM-SA1, RM-SA2,	4.7%	
	*T. orientalis* complex	RM-SA3, RM-SA4)		
	***R. microplus***	1/131 (RM-SA10) 3/131 (RM-SA5, RM-SA6, RM-SA7)	3.1%	80% (8/10)
	*T. velifera*			
	*T. orientalis* complex			
Goat	***R. microplus***	1/131 (RM-SA8)	0.8%	10% (1/10)
	*T. orientalis* complex			
Sheep	***R. microplus***	1/131 (RM-SA9)	0.8 %	10% (1/10)
	*T. orientalis* complex			

### 3.3 Phylogenetic analysis

**Figure 3:**
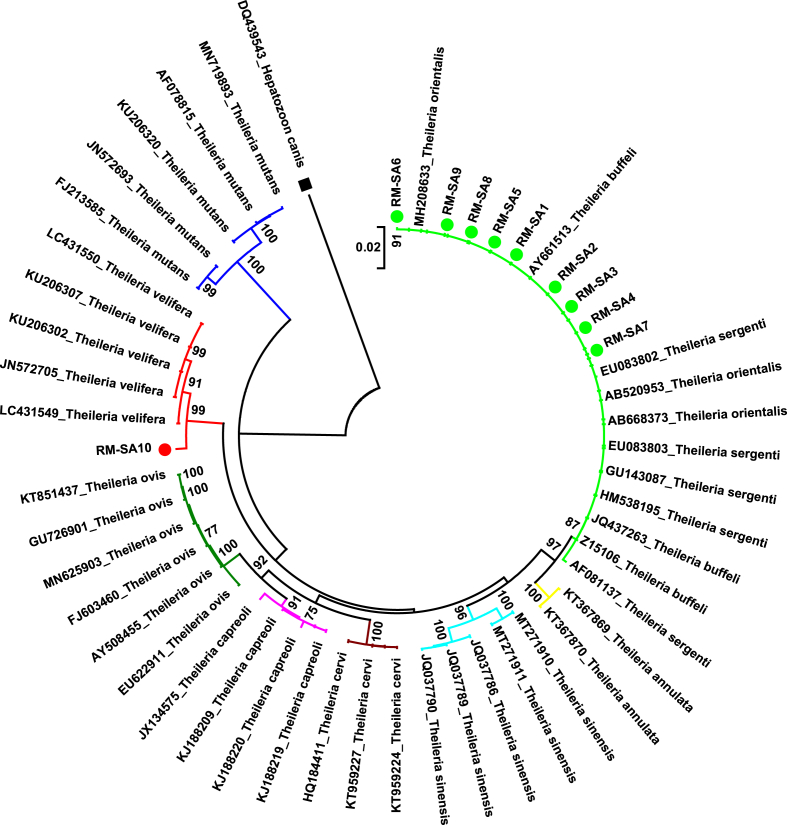
Phylogenetic tree based on partial 18S rRNA gene sequences of *Theileria* spp. The tree was constructed by using the 10 (●) theilerial sequences from this study and 41 additional reference sequences of 10 different *Theileria* species, forming eight major clades as differentiated by the colours, and one (1) sequence of *Hepatozoon canis* (■) as an outgroup. The tree was constructed by using a Neighbor-Joining algorithm as implemented in MEGA 7. The percentage of replicate trees in which the associated taxa formed clusters together in the 1000 replicates bootstrap test are written close to the branches. Only bootstrap values ≥ 70% are shown.

The phylogenetic analysis of the 10 partial 18S rRNA gene sequences of *Theileria* spp. from this study, grouped them into two distinct clades (Figure 3). The majority of the theilerial sequences (RM-SA1 – RM-SA9) clustered within a clade consisting all the *T. orientalis*, *T. buffeli* and *T. sergenti* reference sequences used in the analysis. It could be observed that 10 different species of *Theileria* (from the GenBank) including: *T. orientalis*, *T. buffeli, T. sergenti, T. annulata, T. sinensis, T. cervi, T. capreoli, T. ovis, T. velifera,* and *T. mutans,* were used in the analysis (Supplementary Table 1). However, the *T. orientalis*, *T. buffeli* and *T. sergenti* reference sequences formed a distinct cluster alongside the 9 theilerial sequences from this study, depicting the asserted *T. orientalis* complex (Figure 3). Notably, only the theilerial sequence RM-SA10 in this study clustered alongside other *T. velifera* reference sequences, thereby giving a further confirmation of its identity as earlier suggested by the nucleotide BLAST analysis.

## 4 Discussion

In the present study, occurrence of *Theileria* spp. in ticks was investigated in a total of 962 ticks obtained from livestocks in Raymond Mhlaba local municipality of Eastern Cape Province, South Africa. The overall prevalence of *Theileria* detected was 1% (10/962) in ticks samples from cattle, goats and sheep in one of the study sites (Debe location). This could be due to free-ranging animals in the location, as they were permitted to graze closer to the wild animals, precisely besides the Great Fish nature reserve. However, in Fort Beaufort location where ranching practice is common, animals were restricted in enclosures and therefore remained un-infected. According to Kocan and Kocan (1991), piroplasmid pathogens are frequently associated with livestock and infect free-ranging animals globally. This assertion validated a study in Korea where *Theileria* spp. was confirmed in grazing indigenous cattle with a high infection rate of 54.7% compared to non-grazing cattle (Kim et al., 2017), thus supporting the findings from this study.

Amplification and DNA sequencing of 18S rRNA gene is crucial for the identification of *Theileria* spp. With regards to the species confirmation through the nucleotide BLAST and phylogenetic analysis of the sequenced theileria genomes, the circulating piroplasms in ticks obtained from livestock in the study area have been characterised. A very high identity ranging from 99% for the sequence RM-SA10 with the reference sequence LC431549 (*T. velifera*) from Saudi Arabia, and an 100% similarity recorded for sequences RM-SA1 to 9 with other reference sequences of *T. orientalis, T. buffeli* and *T. sergenti* from different animal hosts and countries, is very significant in terms of global transmission of the pathogen. These findings further suggest that the pathogen is generally circulating between domestic and wild animals across the globe. Within the past decades, trades in animals could have played a huge role in the spread of tick-borne pathogens including the *Theileria* spp. (Perveen et al., 2021), hence, the observed high similarity of theilerial sequences obtained in this study with other reference species from different countries of the world. *Theileria* species are generally known for their severe economic impacts on the livestock industry. The species of *Theileria* detected in this study have been reported in several parts of the world, with significant impacts in game reserve and animal husbandry in general. The first described cases of *T. orientalis* and *T. velifera* was in eastern Siberia (Fujisaki et al. (1994), while *T. buffeli* was first confirmed from the Asian water buffalo as reported by Fujisaki et al. (1994).

Based on molecular data from various studies, *T. buffeli*, *T. sergenti* and *T. orientalis* have been determined to be closely related and therefore have been reclassified as the same species of *Theileria*. Due to similarity in morphology, geographical distribution, serology, vectors involved in transmission, major Piroplasm Surface Protein (MPSP), and 18S rRNA gene sequences, studies have designated these parasites as the *T. orientalis/T. buffeli/T. sergenti* group of benign *Theileria*. Therefore, *T. orientalis/T. buffeli/T. sergenti* is now considered as *T. orientalis* complex (Kamau et al., 2011). From the six species of ticks collected only two species harboured *T. orientalis* complex, the pathogen was specifically detected in *R.eversti eversti* and *R. microplus.* This finding is congruent with that of Kakati et al. (2015) in India, who detected the presence of *T. orientalis* from ten *R. microplus* with an overall infection rate of 83%.

*T. orientalis/buffeli/sergenti* complex previously regarded as a non-pathogenic bovine protozoan species has sporadically lead to severe loss in the livestock industry. Several reports have confirmed the infection of *T. orientalis* in cattle and buffaloes around the world (Kamau et al., 2011; Chaisi et al., 2011; Elsify et al., 2015; Gebrekidan et al., 2017; Nqoro, 2019; Li et al., 2020) and it correlate with results obtained in this study where *T. orientalis* strains were obtained in cattle, sheep and goats. This observation connotes the fact that in the absence of the suitable hosts, they could change their host preferences. This assertion could further be corroborated by the report of Yang et al. (2014) which indicated a high prevalence of *T. orientalis* complex in sheep and goats in China. Also, Elsify et al. (2015) equally detected the existence of *T. orientalis* in cattle, sheep and buffaloes in Egypt, and similarly, strains of *T. orientalis* have been reported in many animals including wild animals such as ungulates and red foxes by Najm et al. (2014). These studies suggest that there is an existence of a common epidemiological cycle amongst wildlife and sympatric domestic animals as regards the theilerial species.

*T. orientalis* has been identified as one of the etiologic agents of bovine theileriosis. While few studies have reported *T. orientalis* infection as asymptomatic in livestock as it causes mild anaemia, recent outbreaks have presented this pathogen as an emerging parasite as supported by various reports of clinical outbreaks characterized by fever, jaundice, anaemia, abortion and even mortality from many countries (Kakati et al., 2015; Gebrekidan et al., 2017). The majority of the cattle infected with *T. orientalis* turned out to become significant chronic carriers of the parasite, but could sometimes sporadically develop severe or fatal anaemia under certain unfavourable illnesses (Kim et al., 2017). Moreover, prenatal infection as a result of intrauterine transmission of *T. orientalis* may be a chronic risk factor that constitute threats to cattle's health from generation to generation (Kim et al., 2017). Much attention has been paid recently to the pathogenicity of *T. orientalis,* as records of fatal disease due to the infection of the parasite in crossbred adult bovines infested with *Haemaphysalis bispinosa* has increased considerably in Southern India (Kakati et al., 2015).

In addition, several epidemiological findings on *T. orientalis* have been reported from many countries including Turkey, France and Spain (Aktas et al., 2006; Altay et al., 2008; Gimenez et al., 2009). In Africa, limited studies have described *T. orientalis* infection in farm animals, however, Moumouni et al. (2015) documented its presence in cattle from Ngong Farm in Kenya while strains of *T. orientalis* were also detected in Ethiopia from domestic animals (Gebrekidan et al., 2016). In South Africa, strains of *T. buffeli* were detected in some Provinces from blood samples of roan antelope with a prevalence rate as high as 100% (Berggoetz et al., 2014). A co-infection of this species with other theilerial species (*T. ovis* and *T. bicornis*) and *Anaplasma* spp. (*A. centrale*, *A. platys* and *A. ovis*) has equally been documented (Berggoetz et al., 2014). Although the incidence of *T. buffeli* is quite low in South Africa, Chaisi et al. (2014) managed to identify *T. buffeli* in buffaloes in Addo park in Eastern Cape Province. Nevertheless, this is one of the few studies reporting the occurrence of *Theileria* species in Raymond Mhlaba local Municipality of the Eastern Cape Province, South Africa.

*T. velifera* is a protozoan pathogen recognised to cause illnesses with symptoms such as enlargement of glands, loss of milk production in cattle and loss of appetite*. T. velifera* has been detected by Githaka et al. (2014) in cattle from Kenya and they reported that it is transmitted by *R. appendiculatus,* an ixodid tick. Similarly, Yusufmia et al. (2010) also identified species of *T. velifera* with 70% prevalence in cattle from KwaZulu-Natal Province of South Africa. Its low detection in this study is highly significant as it could depicts recent transmission of the pathogen to the study area, since it has been previously reported at another Province.

One of the major significant reasons for emergence of these *Theileria* species from other continents to Africa is uncontrollable animal migration, trading, and transportation of animals for economic reasons; although, an increased in human mobility, escalating population growth, and sporadic climate change could possibly constitute major risk factors for geographic expansion of the pathogen to new areas of the sub-Saharan Africa (Perveen et al., 2021). As evidenced by this study, occurrence of *Theileria* in tick species in the study areas of Eastern Cape could become a growing burden on the transmission of new *Theileria* pathogens being introduced and could later become increasingly ubiquitous. And as time goes on, an increased interactions between the wildlife and human due to socio-economic changes could enhance the risk of human beings contracting theileriosis. It is therefore necessary to carry out further sentinel studies in order to monitor the presence of tick-borne pathogens which were not detected in this present study.

## 5 Conclusion

In conclusion, this study has reported the occurrence of *T. orientalis* complex and *T. velifera* species in ticks from cattle, goats and sheep from the Raymond Mhlaba local municipality of Eastern Cape Province South Africa. It is therefore highly recommended that farmers should avoid keeping their livestock in close proximity to their dwellings in order to avoid transmission of tick-borne pathogens to humans. It is necessary to enhance the knowledge about prevention of tick-borne diseases, as it is impossible to eradicate ticks. Frequent dipping of animals is crucial so as to minimize the density and distribution of ticks. Clinicians and Veterinarians should be conversant with the clinical signs of tick-borne diseases and infections among humans and domestic animals respectively. Also, application of quicker diagnostic assays should be considered in a bid to determine the prevalence of tick-borne infections in other rural areas of the country. Additionally, further elaborate studies are recommended in order to precisely detect and distinguish these emerging theilerial species in animal hosts and their relative tick vectors in South Africa.

## Declarations

### Author contribution statement

Benson Chuks Iweriebor: Conceived and designed the experiments; Performed the experiments; Analyzed and interpreted the data; Contributed reagents, materials, analysis tools or data; wrote the paper.

Larry Chikwelu Obi: Conceived and designed the experiments; Contributed reagents, materials, analysis tools or data; wrote the paper.

Ayabulela Nqoro: Performed the experiments; wrote the paper.

Kayode Olayinka Afolabi: Analyzed and interpreted the data; wrote the paper.

### Funding statement

This work was supported by 10.13039/501100001322SAMRC (grant number GL MO8459).

### Data availability statement

Data associated with this study has been deposited at NCBI GenBank under the accession numbers MW647866 to MW647875.

### Declaration of interests statement

The authors declare no conflict of interest.

### Additional information

No additional information is available for this paper.
