# Supplementary material for: Emergence of Theileria species in ticks from free-ranging domestic animals in Raymond Mhlaba local municipality, South Africa
Source: Heliyon. 2022 Mar 10;8(3):e09085. doi: 10.1016/j.heliyon.2022.e09085 (PMC8919220; doi:10.1016/j.heliyon.2022.e09085)
Supplement: Supplementary Table 1 [file mmc1.docx]

**Supplementary Table 1**

**Table 1:** Reference strains of *Theileria* spp. used to construct the phylogenetic tree

| **Accession Numbers** | **Species** | **Geographical origin** |
| --- | --- | --- |
| AB668373 | *T. orientalis* | Japan |
| MH208633 | *T. orientalis* | China |
| AB520953 | *T. orientalis* | Australia |
| JQ437263 | *T. buffeli* | Australia |
| Z15106 | *T. buffeli* | South Africa |
| AY661513 | *T. buffeli* | USA |
| EU083803 | *T. sergenti* | China |
| EU083802 | *T. sergenti* | China |
| GU143087 | *T. sergenti* | Taiwan |
| HM538195 | *T. sergenti* | China |
| AF081137 | *T. sergenti* | China |
| KT367869 | *T. annulata* | India |
| KT367870 | *T. annulata* | India |
| MT271910 | *T. sinensis* | Malaysia |
| MT271911 | *T. sinensis* | Malaysia |
| JQ037786 | *T. sinensis* | South Africa |
| JQ037789 | *T. sinensis* | South Africa |
| JQ037790 | *T. sinensis* | South Africa |
| KT959224 | *T. cervi* | China |
| KT959227 | *T. cervi* | China |
| HQ184411 | *T. cervi* | China |
| KJ188219 | *T. capreoli* | China |
| KJ188220 | *T. capreoli* | China |
| KJ188209 | *T. capreoli* | China |
| JX134575 | *T. capreoli* | China |
| EU622911 | *T. ovis* | France |
| AY508455 | *T. ovis* | Turkey |
| FJ603460 | *T. ovis* | China |
| MN625903 | *T. ovis* | Egypt |
| GU726901 | *T. ovis* | Iran |
| KT851437 | *T. ovis* | Turkey |
| LC431549 | *T. velifera* | Saudi Arabia |
| JN572705 | *T. velifera* | South Africa |
| KU206302 | *T. velifera* | Uganda |
| KU206307 | *T. velifera* | Uganda |
| LC431550 | *T. velifera* | Saudi Arabia |
| FJ213585 | *T. mutans* | South Africa |
| JN572693 | *T. mutans* | South Africa |
| KU206320 | *T. mutans* | Uganda |
| AF078815 | *T. mutans* | Kenya |
| MN719893 | *T. mutans* | Cameroon |
| DQ439543 | *Hepatozoon canis* | Venezuela |
